# Supplementary figures and images for: Somatostatin Receptor Gene Functions in Growth Regulation in Bivalve Scallop and Clam
Source: Int J Mol Sci. 2024 Apr 28;25(9):4813. doi: 10.3390/ijms25094813 (PMC11083992; doi:10.3390/ijms25094813)

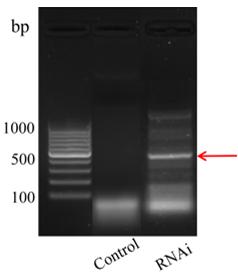

Supplement: Supplementary file 1 [file ijms-25-04813-s001.zip › Figure S1 The detection of dsRNA targeting MlSSTR-1 by agarose gel electrophoresis.jpg]

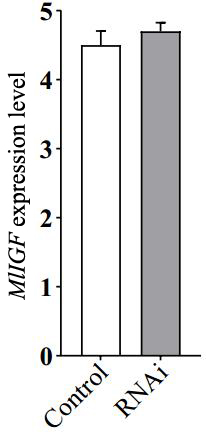

Supplement: Supplementary file 1 [file ijms-25-04813-s001.zip › Figure S2 The expression level of MlIGF in the digestive gland after Ml.SSTR-1 inhibition.jpg]
